# Win-stay-lose-learn promotes cooperation in the prisoner’s dilemma game with voluntary participation

**DOI:** 10.1371/journal.pone.0171680

**Published:** 2017-02-09

**Authors:** Chen Chu, Jinzhuo Liu, Chen Shen, Jiahua Jin, Lei Shi

**Affiliations:** 1 School of Statistics and Mathematics, Yunnan University of Finance and Economics, Kunming, Yunnan, China; 2 School of Software, Yunnan University, Kunming, Yunnan, China; 3 Library of Yunnan Normal University, Kunming, Yunnan, China; Beihang University, CHINA

## Abstract

Voluntary participation, demonstrated to be a simple yet effective mechanism to promote persistent cooperative behavior, has been extensively studied. It has also been verified that the aspiration-based win-stay-lose-learn strategy updating rule promotes the evolution of cooperation. Inspired by this well-known fact, we combine the Win-Stay-Lose-Learn updating rule with voluntary participation: Players maintain their strategies when they are satisfied, or players attempt to imitate the strategy of one randomly chosen neighbor. We find that this mechanism maintains persistent cooperative behavior, even further promotes the evolution of cooperation under certain conditions.

## Introduction

The emergence and stability of cooperation among unrelated selfish individuals has become a major challenge in biology, economic, and behavioral sciences [1–3]. In order to study this issue, evolutionary game theory has become a useful tool [4]. In particular, a simple model, the prisoner’s dilemma (PD) game, was considered as a paradigm [5]. In its basic version, two players concurrently decide to take one of two strategies: cooperation (C) and defection (D). They will receive the reward *R* if both cooperate and the punishment P if both defect. However, if one player defects while the other decides to cooperate, the former will get the temptation T while the latter will get the sucker’s payoff S. The payoffs are ordered as *T* > *R* > *P* > *S* so that in the well-mixed case defection is the best strategy regardless of the opponent strategy.

In the pioneering work by Nowak and May [6], spatial games were introduced, where players are arranged on the spatially structured topology and interact only with their direct neighbors. Thus the topology has become a determinant for the success of cooperative behavior (regular networks [7, 8], small world networks [9–11] and scale-free networks [12–14], multilayer network [15–17]). Other approaches facilitating the evolution of cooperation include environment [18–21], payoff [22–25], features of players [26–29] and so on (for a comprehensive understanding referring to Ref. [30, 31]). Besides, voluntary participation has recently been demonstrated to be a simple yet effective mechanism to promote persistent cooperative behavior [32, 33]. The introduction of loner strategy, someone may do not willing to participate in the PD game and would rather take a small but fixed payoff, can lead to a rock-scissors-paper cyclic dominance that means the cooperation can still survive even with large temptation of defection [33]. In these works, individual players are assumed to update their strategies by learning from their neighbors in almost every round of game. Meanwhile, for reality, the win-stay rules [34–39] are introduced, which assume individual players only change their strategies when they feel dissatisfied. These rules are proved to be a robustly mechanism that can promote the evolution of cooperation independently of the initial conditions. Naturally, an interesting question is here posed: how cooperation fares when the win-stay updating rule is introduced into PD with voluntary participation.

In this paper, we combine the win-stay-lose-learn strategy updating rule with voluntary participation. The so-called win-stay-lose-learn strategy updating rule is designed as follows. Players maintain their strategies when they are satisfied, or players attempt to imitate the strategy of one randomly chosen neighbor. Our main aim is to study the impacts of such a mechanism on the spreading of cooperation. Through numerical simulations, we show that such a scenario can maintain persistent cooperative behavior, even further promotes the evolution of cooperation under certain conditions.

## Results

**Fig 1:**
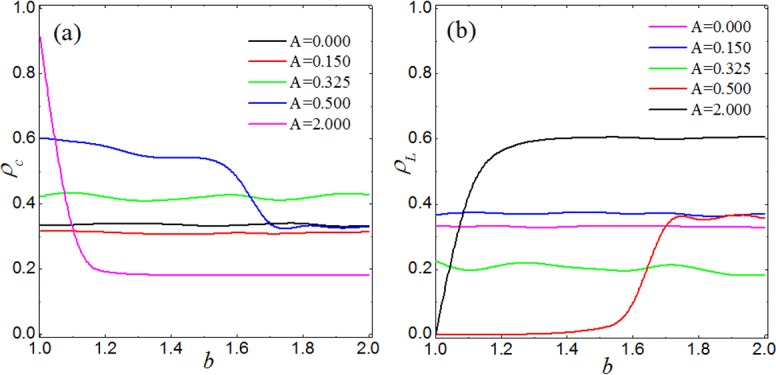
**Average fractions of cooperation (panel (a)) and loners (panel (b))** as a function of *b* for different values of the aspiration *A*, as obtained by means of simulations on square lattices.

We start our result with the case where cooperators, defectors and loners are distributed uniformly at random, each thus initially occupying third of the square lattice. As the main parameters, we consider the aspiration level *A* and the temptation to defect *b*. Fig 1 (A) shows the fraction of cooperation *ρ*_*c*_ as a function of the temptation to defect *b* for different aspiration levels *A*. We find that the aspiration level has a significant influence on the evolution of cooperation. As shown in Fig 1, the fraction of cooperation decreases slowly with increasing *A*, but the temptation to defect has no effect on cooperation, e.g., for *A* = 0 and *A* = 0.15, *ρ*_*c*_ = 0.33 and *ρ*_*c*_ = 0.31, respectively, irrespective of the value of *b*. When 0.3 < *A* ≤ 0.5, the fraction of cooperation decreases as *A* increases. In addition, as *b* increases, transitions to different stationary states can be observed for certain values of *A*. For example, the break point occurs at *b* = 1.7 for *A* = 0.5, which will be explained below. When 0.5 < *A* < 0.75, highest levels of cooperation occurs if the value of *b* is larger than 1.2 around. When *A* > 0.75, the fraction of cooperation is maintained at 0.18. Last, it is worth pointing out that, when *A* is large (*A* = 2.0), individuals are always dissatisfied with their payoffs, our model then returns to the traditional version of prisoner’s dilemma game. Based on the above results, the present updating rule can effectively facilitate the evolution of cooperation (except for *A* = 2.0, cooperation can survive only if *b*<1.05). By contrast, with joint interaction of voluntary participation and win-stay-lose-learn updating, cooperation can not only survive but also thrive in the case of larger *b*.

**Fig 2:**
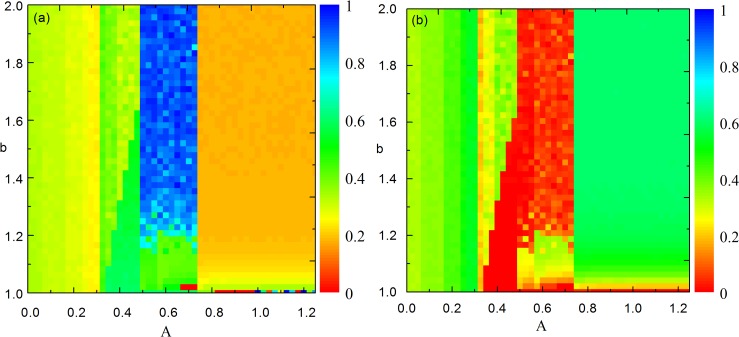
Color-coded (see bar on the right) fraction of cooperation and loners. The multitude of transitions in the color map points towards a high complexity of the underlying mechanisms (see main text for more details).

In order to obtain a more complete picture about the joint effects of the aspiration level and the temptation to defect, we show the simulation results as a function of both *A* and *b*. As shown in Fig 2, the results are consistent with those presented in Fig 1(A), e.g., when *A* < 0.3, the fraction of cooperation decreases discontinuously with increasing *A*, irrespective of the value of *b*. when *A* = 0.3, the cooperation level recovers to 0.4, and then decreases discontinuously with increasing *A* until *A* = 0.5. At the same time, the transition can be observed at a fixed value of *b* for each value of *A*. It is interesting that the highest level of cooperation occurs within the interval of 0.5 < *A* < 0.75 when *b* > 1.2. Moreover, it can be observed that as *A* increases, discontinuous transitions occur at *A* = 0.0,0.075,0.15,0.225,0.3,0.5,0.75. These transitions can be explained as follows. On a square lattice with nearest neighbor interactions, the payoffs of a cooperator, a defector and a loner are given by *n*_1_*R* + *n*_2_*S* + *n*_3_*σ*, *n*_4_*T* + *n*_5_*P* + *n*_6_*σ* and 4*σ*, respectively, where *n*_*k*_ ∈ {0,1,2,3,4}, and *k* ∈ {1,2,3,4,5,6}. Given that *T* = *b*, *R* = 1, *P* = *S* = 0, and *σ* = 0.3 the above payoffs can be simplified as *n*_1_ + 0.3*n*_3_, *n*_4_*b* + 0.3*n*_6_ and 1.2, respectively. In our model, when an individual is dissatisfied, it will learn from a randomly chosen neighbor, which may lead to the change of fractions of cooperation and loners. For a cooperator, when *n*_1_ + 0.3*n*_3_ < 4*A*, it is dissatisfied. While for a defector, the condition for its dissatisfaction is *n*_4_*b* + 0.3*n*_6_ < 4*A*. And for a loner, it will dissatisfy when 4*A* > 1.2. The phase transition points can be obtained by letting *n*_1_ + 0.3*n*_3_ = 4*A* and *n*_4_*b* + 0.3*n*_6_ = 4*A*. Thus, the value of *A* at which phase transition occurs is given by *A* = (*n*_1_ + 0.3*n*_3_)/4 and that for *b* is given by $b=\frac{4A-0.3n_{6}}{n_{4}}>1$. Considering all the possible values of *n*_1_ and *n*_3_, that is *n*_1_ = 0,1,2,3,4 and *n*_3_ = 0,1,2,3,4, we can obtain the phase transition points of *A*, which are *A* = 0,0.075,0.15,0.225,0.25,0.3,0.325,0.4,0.475,0.5,0.575,0.65,0.75,0.825,1. As a matter of fact, the phase transition phenomenon can't be observed when *A* = 0.825 and 1 because of the low fraction of cooperation.

**Fig 3:**
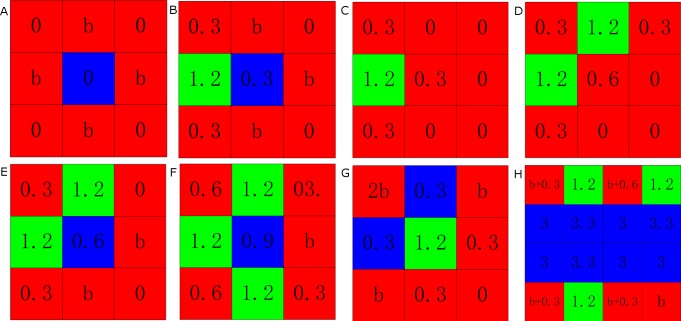
Special configurations of players. In all panels the cooperators are depicted blue while defectors are depicted red and loners are depicted green. Each small square corresponds to a single player. Denoted values correspond to the payoffs of individual players, as obtained for the presented configurations.

**Fig 4:**
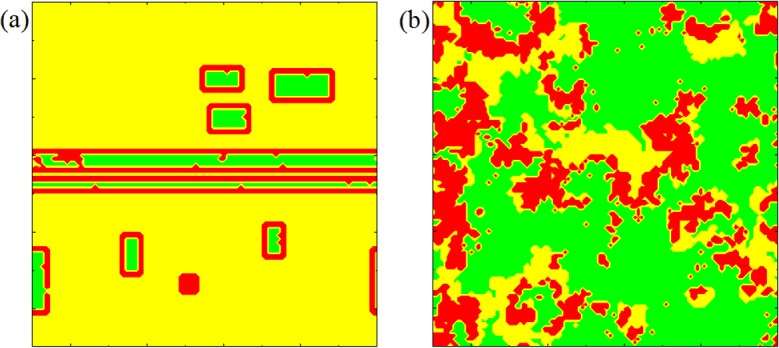
Evolution of cooperation on stable state under some certain initial condition. Panel (a) (or (b)) features the characteristic snapshots of the spatial grid when A = 0.75 (A = 0.751) and b = 1.5, as obtained when using the initial conditions presented in Fig 3(H) (cooperators are yellow, defectors are red and loners are green).

The configuration of players are relevant for the evolutionary success of cooperation in spatial games [40–42], it is also of interest to test the robustness of the proposed updating rule. We thus investigate how cooperation evolves under different (adverse) configurations. When 0 ≤ *A* < 0.075, only cooperators or defectors surrounded by defectors [see Fig 3(A)] are dissatisfied. The cooperator surely resorts to defection by imitation because of his dissatisfaction, while the defector has to remain his strategy. Hence, the fraction of cooperation will decrease while defectors increase. When 0.075 ≤ *A* < 0.15, the cooperator or the defector next to a loner [see Fig 3(B) and Fig 3(C)] is dissatisfied. The cooperator will choose defection or turn to a loner, and the defector would probably become a loner. Thus the fraction of cooperation will decrease while loners increase. For 0.15 ≤ *A* < 0.225, defectors and loners next to two loners [see Fig 3(D) and Fig 4(E)] want to change their strategies. Trends in fractions of three kinds of players are similar to previous situations. While 0.225 ≤ *A* < 0.3, cooperators and defectors whose neighbors include three loners [see Fig 3(F)] begin to feel dissatisfied. As a result, the fraction of cooperation decreases while loners increase. When *A* > 0.3, the payoff of loners is 1.2 that is less than 4 * *A*. Thus, all loners [see Fig 3(G)] are dissatisfied such that they imitate neighbors’ strategies. This is the reason why we can observe that the fraction of loners falls suddenly at *A* = 0.3. When < 0.3*A* ≤ 0.5 and *b* < 4 * *A* − 0.3 * *n*, where *n* is the number of risk averse neighbors, players that has one cooperative neighbor are dissatisfied. As dissatisfied players avoid being loners, the fractions of cooperation and defection remain stable. When *b* > 4 * *A* − 0.3 * *n*, defectors that has one cooperative neighbor become satisfied. Then the Rock–Scissors–Paper-type cycles occur because of the advantages of defectors. The more favorable case emerges when 0.5 < *A* < 0.75. Within the interval of 0.5 < *A* < 0.75, cooperators that have three cooperative neighbors can maintain their strategies and very likely form clusters [see Fig 4(A)] which ensure their advantages over defectors and loners in term of payoffs. Then they expand the area of cooperation. Meanwhile, defectors that have at least two cooperative neighbors are satisfied, and at last surrounded by cooperators. This is the reason why the highest levels of cooperation occur within the interval of 0.5 < *A* ≤ 0.75. When 0.75 < *A*, satisfied players including cooperators that have four cooperative neighbors and defectors that have at least [3/*b*] + 1 cooperative neighbors as shown in Fig 4(B) cannot maintain stable states. This cyclic Rock-Scissors-Paper type of dominance determines the system’s dynamics again. The fractions of three kinds of players are similar to those of normal prisoner’s dilemma games with voluntary participation.

## Conclusion

To conclude, we have studied the impact of the win-stay-lose-learn strategy updating rule on the evolutionary prisoner’s dilemma game with voluntary participation. The risk averse loner strategy (*L*) is introduced and the strategies depend on the level of satisfaction of individuals. We find that the new rule is able to maintain persistent cooperative behavior, even further promotes the evolution of cooperation under certain conditions. The win-stay-lose-learn rule dominates the level of cooperation when individuals are not too greedy. It restrains the individual players’ impulses of changing their strategies with small aspiration levels (*A* ≤ 0.5), while it promotes the evolution of cooperation especially for intermediate values of the aspiration parameter (0.5 < *A* ≤ 0.75), virtually complete cooperation dominance can be achieved even for values of the temptation to defect that significantly exceed 1. This is in sharp contrast to the results obtained with large aspiration levels (*A* > 0.75), where the cyclic rock-scissors-paper type of dominance is essentially fully recovered. Compared with previous works, our model considers both two points so that the new rule can promote the evolution of cooperation for intermediate values of the aspiration parameter, and maintain persistent cooperative behavior with large aspiration levels. Our work is expected to provide a valuable method that can resolve the prisoner’ dilemma.

## Methods

We consider voluntary participation and a win-stay-lose-learn strategy updating rule in the PD on a square lattice of size *L*^2^ with periodic boundary conditions. The risk averse player is defined as the loner (*L*). Loners and their co-players always obtain the fixed payoff 0 < σ < 1 that makes their overall payoffs more than that of two defectors but are less than that of cooperative pairs. The aspiration level of a player is defined as *A*_*i*_ = *K*_*i*_ * *A*, where *K*_*i*_ is the player’s degree and *A* is a free parameter of the average aspiration level within the interval of (0, *b*). Following a common practice [6], we choose the PD’s payoffs as *R* = 1, *P* = *S* = 0, and *T* = *b*>1, satisfying the restricted condition *T*>*R*>*P* = *S*.

We implement the evolutionary dynamics in the following way. As initial conditions, we assign to each individual, with equal probability, one of the three available strategies: cooperation (C), defection (D) or lone (L). Then, at each time step, each player *i* in the network obtains the payoff *P*_*i*_ by playing with all its neighbors. Next, all the players synchronously update their strategies by comparing the respective payoffs *P*_*i*_ and *A*_*i*_. If *P*_*i*_ > *A*_*i*_, player *i* will keep its strategy for the next step. On the contrary, if *P*_*i*_ < *A*_*i*_, player *i* will pick up at random one of their neighbors, say *j*, and adopt *j*’s strategy with the probability:

$$W=\frac{1}{1+exp[(P_{i}-P_{j})/K]}$$

where *K* stands for the amplitude of noise [43–46]. Without loss of generality, we use *K* = 0.1 for the PD. To assure that the system has reached a stationary state we make the transient time t equals 100000. Then we can obtain the presented results by using *L* = 100 system size. Moreover, each data were averaged over up to 20 independent runs for each set of parameter values in order to assure suitable accuracy.

## Supporting information

S1 File This file (Zip format) contains the raw data used in figures with MC simulation. (ZIP)
